# Designed Synthesis of Three-Dimensional Covalent Organic Frameworks: A Mini Review

**DOI:** 10.3390/polym15040887

**Published:** 2023-02-10

**Authors:** Pham Thi Phan, Qui Thanh Hoai Ta, Phan Khanh Thinh Nguyen

**Affiliations:** 1 Faculty of Food Science and Engineering, Lac Hong University, Bien Hoa 810000, Vietnam; 2 Department of Physics, Gachon University, 1342 Seongnamdaero, Sujeong-gu, Seongnam-si 13120, Gyeonggi-do, Republic of Korea; 3 Department of Chemical and Biological Engineering, Gachon University, 1342 Seongnamdaero, Sujeong-gu, Seongnam-si 13120, Gyeonggi-do, Republic of Korea

**Keywords:** covalent organic frameworks, building blocks, octahedral units, growth mechanism

## Abstract

Covalent organic frameworks are porous crystals of polymers with two categories based on their covalent linkages: layered structures with two dimensions and networks with three-dimensional structures. Three-dimensional covalent organic frameworks are porous, have large surface areas, and have highly ordered structures. Since covalent bonds are responsible for the formation of three-dimensional covalent organic frameworks, their synthesis has been a challenge and different structures are generated during the synthesis. Moreover, initially, their topologies have been limited to dia, ctn, and bor which are formed by the condensation of triangular or linear units with tetrahedral units. There are very few building units available for their synthesis. Finally, the future perspective of 3D COFs has been designated for the future development of three-dimensional covalent organic frameworks.

## 1. Introduction

Covalent organic frameworks (COFs), a branch of a crystalline framework with pores, are synthesised by combining directional organic fragments covalently to form reticular polymers. They are polymers having specific morphologies and geometrical conformations [1,2,3]. They create a restrained molecular area and interface for the functioning of electrons, photons, holes, excitons, molecules, and ions. COFs are formed by the growth of polymers from monomers having similar geometry [1].

The principle of polymer growth is controlled by a topology diagram that is different from the principle used for polymers such as cross-linked polymers, linear polymers, biopolymers, and hyperbranched polymers; thus, highly ordered structures can be predesigned at the primary stage [4,5,6]. The molecular building blocks combine covalently with each other to form either a two-dimensional (2D) or three-dimensional (3D) geometry with atomic accuracy [7,8,9,10].

Since the propelling force for the synthesis of 3D COFs is the formation of covalent bonds, it poses a difficulty for their synthesis [9]. In contrast, it is comparatively easier to construct a 2D COF as it has multiple noncovalent interactions. Additionally, the orientation of covalent bonds towards a specific direction at accurate angles is an additional difficulty for their synthesis of 3D COFs [10]. Hence, rigid building blocks are favoured for their synthesis [9].

The functional and structural diversity has been explored for different rigid building blocks to predict their geometry based on the restricted rotation of the building blocks. A process of reversible dynamic equilibrium is feasible because of the restricted rotation [2]. There has been a remarkable development of COFs since they were reported in 2005 [3]. The functionality of frameworks [4], low densities, good stability [5,6], high flexibility [7], and pore size modulation [8] are certain remarkable properties of COFs for which they have multiple applications: molecular separation,[9] photocatalysis [10,11], advanced organic materials for electrodes [12], cancer therapeutics [13], modulated materials for electrocatalysis [14], biomedical applications [15], and membrane separation [16].

There are multiple reviews on porous COFs [17], engineering the pore surfaces of COFs, and synthesis of 3D COFs [18,19,20]. Apart from the thermodynamically favoured solvothermal synthesis mainly preferred for the preparation of 3D COFs, there are very few conveyed synthetic methods. However, we found that there are no recent reviews encompassing the comprehensive growth mechanism of 3D COFs. We have put a detailed discussion on the following topics in this review: growth mechanisms, different topologies, and promising applications of 3D COFs. We have concluded our review with a conclusion and future outlook of 3D COFs with both rigid and flexible building blocks.

## 2. Principles of Reticular Chemistry

A particular net or topology of a COF can be aimed at using reticular chemistry. Building blocks of the desired geometry are selected and condensed to place them in a particular framework [19]. There are fi steps in the reticular synthesis of COFs:

(1) The desired framework of the topology is selected and the framework is divided by slicing the edges into vertices;
(2) The geometry, accurate angles, and vertices are analysed depending on the number of points required for extension;
(3) Identification of molecular equivalents of vertices is conducted by selecting huge polyaromatic molecules as building blocks as an accurate geometry can be predicted with the rigid linkers;
(4) The building blocks are connected through covalent linkages to create COFs by modulating the reaction kinetics and reversibility at a microscopic scale between the building units;
(5) The reticulated structure should be analytically confirmed. The accurate architecture of the vertices should be clear since topologies depend on the type of connectivity.

The 3D COFs are targeted by using two different tactics: (a) the topology is allowed to form by assembling supramolecules through a controlled alignment of the building units and (b) the application of polyhedral organic units, mainly organic cages, as building blocks [20]. In the first tactic, the topology is not prefabricated.

The flexibility in the conformation of the organic bonds is responsible for dictating the orientation of the building blocks [21]. Since the flexibility of conformation is challenging to regulate, a specific topology cannot be expected by this approach. In the second tactic, the building units (octahedral units [22], trigonal prism units [23], and tetrahedral units [24]) are predesigned and allowed to combine to form the desired COFs. Thus, a polyhedral geometry having rigid linkers is used as building blocks that are reticulated to create a 3D architecture. These predetermined linkers increase the probability of acquiring the desired geometry. For instance, frameworks with a dia topology are obtained by combining linear and tetrahedral building blocks, whereas a combination of square planar and tetrahedral building entities form frameworks with a pts topology. Trigonal tritopic and tetrahedral building entities integrate to form ctn and bor nets [25].

## 3. Growth Mechanism of 3D COFs Using Different Synthetic Methods

### 3.1. Homogeneous Synthetic Condition

Amorphous polyamine is initially precipitated during the conventional synthesis of imine-based 3D COF in the presence of aqueous acetic acid as a catalyst. This heterogeneous mixture is converted to crystalline COFs by extended heating. Since the growth and nucleation of COFs are heterogeneous, their morphology is similar to the polyimine precipitate. This limitation was removed by preparing 3D COFs under a homogeneous condition and discarding the heterogeneous condition. Thus, a protected amine, 4-(tert-butoxycarbonylamino) aniline (NBPDA) using *tert*-butyloxycarbonyl (Boc) group was used as the starting component. Deprotection of amine was conducted in situ in the presence of trifluoroacetic acid; this process boosted the crystallization of COFs by decreasing the reaction rate of the condensation reaction. The formation of insoluble amorphous intermediates was eradicated, and a woven 3D COF (3D COF-woven) was formed. The protected amine was allowed to form a Schiff base diiminopyridine (DIP) by reacting with Co(BF_4_)_2_·6H_2_O at ambient temperature. The formation of molecular threads is propelled by the metal complex for complete crystallization [26]. The protected amine creates a homogeneous route for the coordination reaction between the ligand and metal before the formation of the polyimine chain. The Schiff base forms an intermediate that can be isolated and is not simply an amine building block. The crystallites obtained were aggregated and small.

### 3.2. Purification of the Amorphous State

The formation of the 3D COF linked by imine bonds was studied using COF-300 as a model compound [27]. The evaluation of the structure remains a challenge, although an adamantine-like structure can be forecasted for the 3D crystal obtained from terephthaldehyde and tetrakis(4-aminophenyl)methane (TAPM). One-dimensional pores are present in the highly interpenetrated form of COF-300. The collapsed form of crystalline COF-300 has a low surface area and was obtained by heating the monomer at 90 °C under atmospheric pressure for 48 h. The porous form could not be obtained from the collapsed form of the crystal through activation methods like high vacuum elimination at a high temperature. The synthesis of the crystal in the absence of water also formed the collapsed structure as the structure gets altered by the water eliminated during the formation of imine. The water binds strongly within the crystal structure and reduces the surface area. The addition of the monomers forms amorphous solids which were purified and reheated at 90 °C under atmospheric pressure for 48 h without further addition of monomers. Thus, porous crystals with a large surface area were obtained proving the fact that purification of the amorphous compound initiates the crystallization process. Water is eliminated through the purification process which is mandatory for the formation of porous COF-300. The contribution of the three solvents was essential for the conversion of the amorphous form into the crystallised form: water, 1,4-dioxane, and acetic acid. Additionally, the exchange of imine is also essential for triggering the crystallization process. The geometry of the amorphous solid strongly directs the formation of the crystalline structure during the rearrangement of imine. The porous COF-300 fraction was enhanced under biphasic conditions than under monophasic conditions. The surface area of the crystal increases six-fold with an increase in the amount of the porous form. However, the isolated and purified amorphous solid gave the maximum yield of the porous COF-300. Hence, modulation of the amorphous intermediate is the key to obtaining porous crystalline COFs.

### 3.3. Maintenance of Thermodynamic Equilibrium

**Figure 1:**
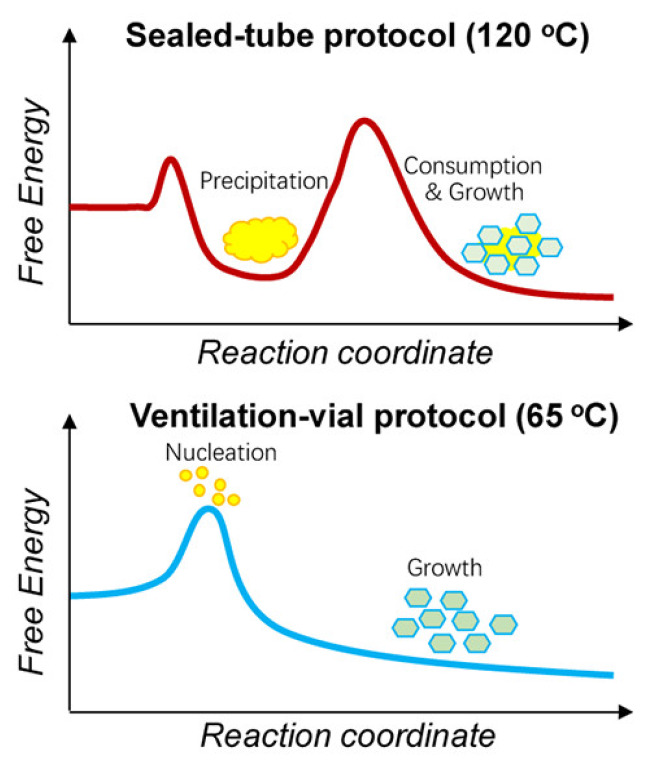
Two different processes for the synthesis of crystals. Reprinted (adapted) with permission from [28]. Copyright 2019 American Chemical Society.

In another work, a scalable synthesis of COF-300 was conducted to explore the dynamics for obtaining crystalline COFs [28]. The standard method of preparing microcrystalline COFs is by maintaining a thermodynamic equilibrium by creating a closed system within a sealed Pyrex tube. High temperature and freezing are used for converting the initially formed amorphous structure to a crystalline structure as shown in Figure 1.

Thus, multiple phases are obtained as it is stringent to govern the morphology of the synthesised compounds. COF-300 was prepared using the ventilation-vial protocol by producing a polarity or acidity gradient. Hence, the morphology of the crystal is governed by a process of homogeneous nucleation to obtain highly crystalline products. The temperature of the system should be low to prevent the oxidation of anilines, and the required amount of water should be available within the system for making the imine-condensation reaction reversible. At the same time, the process of nucleation following the growth of crystals should be modulated by reducing the solubility. The reaction was conducted at 65 °C and the crystallinity was controlled by using a suitable solvent, 1,4-dioxane. The acidity of the system is boosted by decreasing the solubility using 1,4-dioxane. The addition of water molecules contracts the crystal by forming hydrogen bonds within the channels, whereas the addition of THF, cyclohexane, and 1,4-dioxane expanded the crystal volume due to framework displacement, alteration of the geometry of nodes, and alteration of the organic linker configurations (torsion angles, bond angles, and distances between nodes). Conversely, the elimination of THF contracts the crystal. Thus, THF can be used for expanding and contracting COF-300. Similar expansion and contraction were observed with the adsorption of gases like carbon dioxide, methane, argon, and nitrogen. A domino effect is responsible for the geometric alterations due to the interpenetrable nature of the COF.

### 3.4. Continuous Flow Method

**Figure 2:**
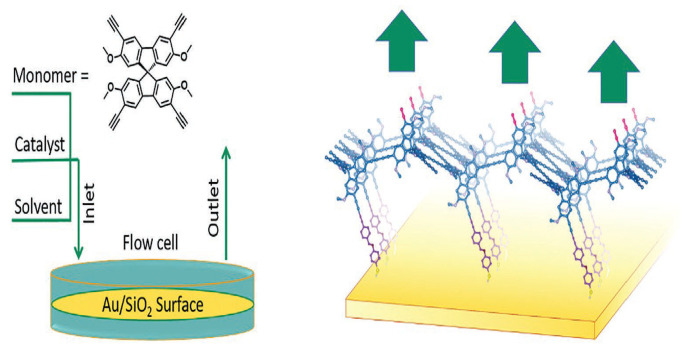
Synthesis of 3D COF films by continuous flow method [32].

Reversible reactions are essential for the synthesis of 3D COFs by solvothermal synthesis. The reagents are heated and allowed to react over a certain period so that nitrogen- and boron-based linkers, having reversible bonds, can dissociate and reform to form a thermodynamically stable crystalline structure [29]. The powdered form of individual crystals is obtained which hinders the exploration of their intrinsic characteristics [30]. The thermodynamically stable state of the synthesised COFs cannot be attained by the solvothermal process as it is prevented by the not-so-dynamic carbon–carbon bonds. The bonds are controlled during the formation of COFs using a continuous flow method where the reaction occurs on the surface of a template [31]. A chemical handle is created for the COF using the surface of a monolayer formed by self-assembly. The reactants bind in the same plane to form the first layer. The amount of free functional groups is high on the second layer formed on top of the first layer. The functional groups of the reactants in the second layer, already bound to the first layer, interact further with the layer by forming bridges between the functional moieties. The first two layers get locked to form a template for directing the crystal to grow upward in a particular direction. 3,3″,6,6″-tetraethynyl-2,2″,7,7″-tetramethoxy-9,9′-spirobifluorene (SBFyne) was allowed to couple in the presence of the CuI catalyst to form a conjugated COF film [32]. The terminal alkyne of 3,3″,6,6″-tetraethynyl-2,2″,7,7″-tetramethoxy-9,9′-spirobifluorene reacts with the alkyne groups on the self-assembled monolayer to form a tetrahedral geometry and flow above the first layer on Au surface. The reaction follows the first-order kinetics since the surface area remains constant. Additionally, the undesired products of coupling are eliminated in the bulk phase which has the probability to follow second-order kinetics. The reaction is controlled by modulating the reagent concentration. The reaction becomes faster at the surface with a lower concentration. The roughness of the surface can be diminished by decreasing the concentration of monomers. Butadiynyl linkers were formed by the terminal alkyne groups and the concentration of defects in the terminal alkyne is less. The formation of 3D COF films on the Si surface was devoid of any defects and had strong integrity. The roughness of the surface on which the film is allowed to grow plays a vital role in the roughness of the fabricated films. A smooth film was obtained by this process as compared to that of the conventional approach. The formation of an organic 3D network was proved by the presence of diffraction peaks with small angles. Although the COF films were insulators, they were p-doped by 7,7,8,8-tetracyanoquinodimethane (TCNQ) and transformed into conductive layers and were examined as active layers in electrochemical transistors with good results. Transfer of charges takes place through the π-conjugated bonds as shown in Figure 2.

### 3.5. Tuning the Linkers to Obtain a Predefined Topology

**Figure 3:**
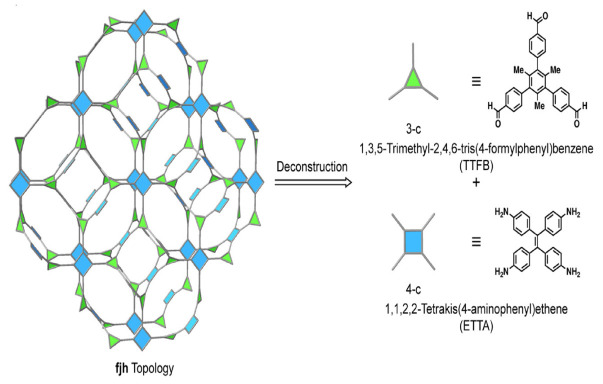
Synthesis of 3D COF with fjh topology. Reprinted (adapted) with permission from [34]. Copyright 2020 American Chemical Society.

The trigonal linkers were modulated for designing a 3D COF with an fjh topology. Since the building units required for the fjh topology were square-planar and triagonal blocks with dihedral angles within a range of 75–90° [33], 1,1,2,2-tetrakis(4-aminophenyl)ethene (ETTA) and 1,3,5-trimethyl-2,4,6-tris(4-formylphenyl)benzene (TTFB) were combined to form a framework with imine linkage and the fjh topology as shown in Figure 3 [34]. A dihedral angle within the desired range is formed by the methyl groups in 1,3,5-trimethyl-2,4,6-tris(4-formylphenyl)benzene. Hence molecular models were prepared which showed dihedral angles of 90°, 83°, and 74°. Thus, a highly crystalline COF-790 was obtained by the solvothermal process.

Interpenetration is another characteristic affecting the formation of 3D COFs [35]. This occurs due to extra growth of the framework in the voids present within the skeleton. Interpenetration affects the porosity of crystals negatively and should be reduced [36].

### 3.6. Steric Hindrance Method

A steric hindrance method was used for the synthesis of 3D mesoporous COFs (3D COF-meso) having a non-interpenetrated dia topology [37]. The pore size of the COF was modulated by controlling the methyl substituents of methoxy-modified monomer, 2,2′,7,7′-tetramethoxy-9,9′-spirobi[fluorene]-3,3′,6,6′-tetracarbaldehyde (TMSFTA), a tetrahedral building block. 4,4′-diaminobiphenyl (DABP) and two of its derivatives with 2 methyl substituents and 4 methyl substituents were used as bridging units to design three different COFs.

**Figure 4:**
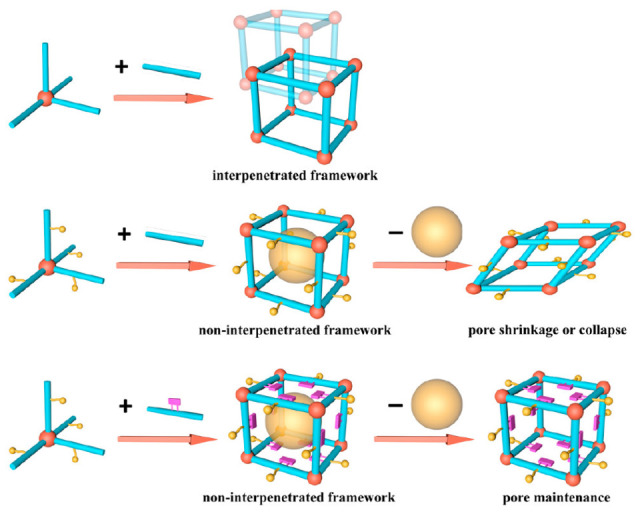
Different possibilities to create 3D COFs by steric hindrance method. Reprinted (adapted) with permission from [37]. Copyright 2020 American Chemical Society.

There are different possibilities to control the morphology of 3D COFs by steric hindrance process: (a) the partially formed edges may create the non-interpenetrated porous framework where the pores may collapse on the elimination of the guests, (b) the randomly formed edges may initiate the formation of an interpenetrated porous structure, and (c) the formation of a non-interpenetrated porous arrangement where the pores are retained even after the elimination of the guests as shown in Figure 4.

The COF (JUC-550) with 4,4′-diaminobiphenyl as bridging units, devoid of methyl substituents, exhibited reversible alteration of the crystal structure in the presence of guests. However, the methyl-substituted 4,4′-diaminobiphenyl as linkers created COFs- JUC 551 (with two methyl substituents) and JUC 552 (with 4 methyl substituents) which exhibited strong rigidity, abstaining dynamic nature of the structure. The high rigidity of the skeleton is attributed to the steric hindrance by the methyl substituents. Thus, the amount of substituents present in the linker is responsible for the formation of stable mesopores. The mesoporous COFs showed good uptake of Rh6G, a dye of high molecular weight.

### 3.7. Imine Exchange Strategy

**Figure 5:**
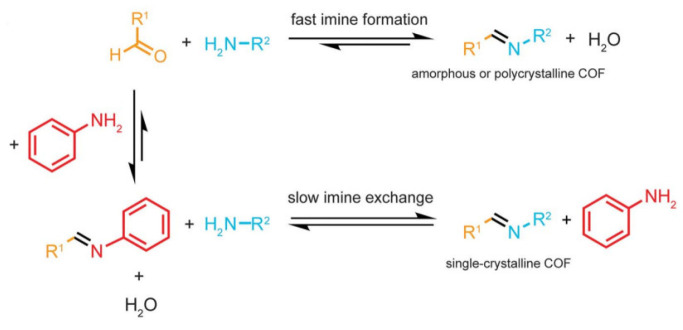
Aniline as a modulator for controlling the reversibility of the reaction [38].

The synthesis and crystallization of 3D COFs were conducted by enhancing the reversibility of the formation and breakage of imine linkage by introducing aniline as a modulator. The reactivity of aniline is similar to the tetrahedral building units, silane-based tetra-aldehyde (TFS), and methane-based tetraamine (TAM) [38]. Aniline was a better modulator than the other substituted aromatic amines. Aniline inhibits nucleation selectively, and amines attack the imine bonds as a strong nucleophile. The amount of amine is elevated in the reaction medium after the addition of aniline, boosting the correction of errors and reversibility of the mechanism of the formation of linkers as shown in Figure 5. A single crystal 3D chiral COF LZU-111 was obtained in the presence of aniline. The crystal has a lonsdaleite network of threefold interpenetrated structures.

**Figure 6:**
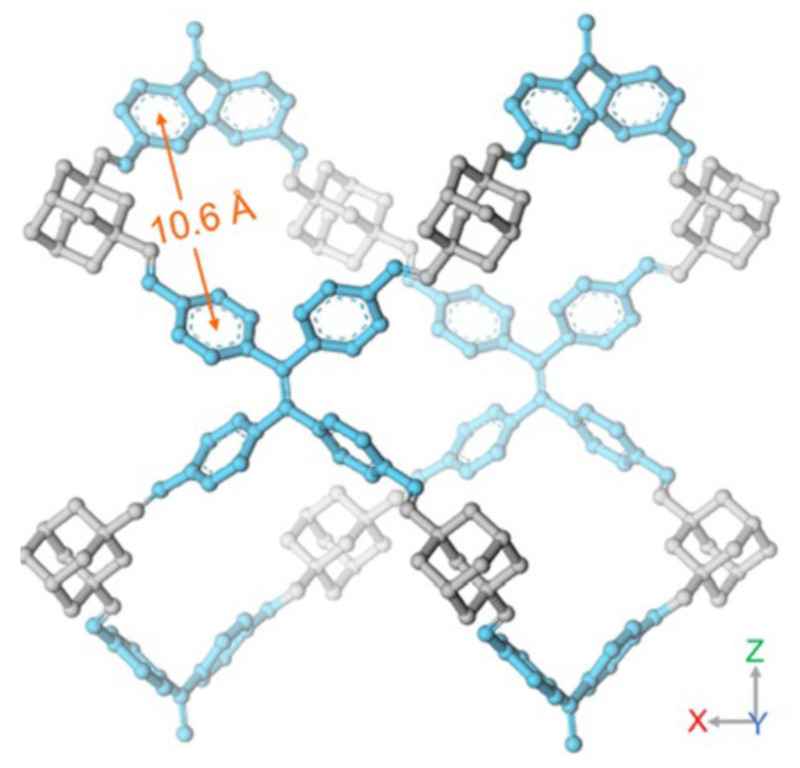
Crystal structure of LZU-306 [39].

In another study, amine as a modulator was used for the synthesis of a 3D single crystal (LZU-306) with a non-interpenetrated pts topology employing adamantine (ADA) as a tetrahedral node and tetraphenylethylene (TPE) as a quadrilateral linker [39]. The quadrilateral linkers are segregated evenly by the adamantine units as shown in Figure 6.

### 3.8. The Partition Method

**Figure 7:**
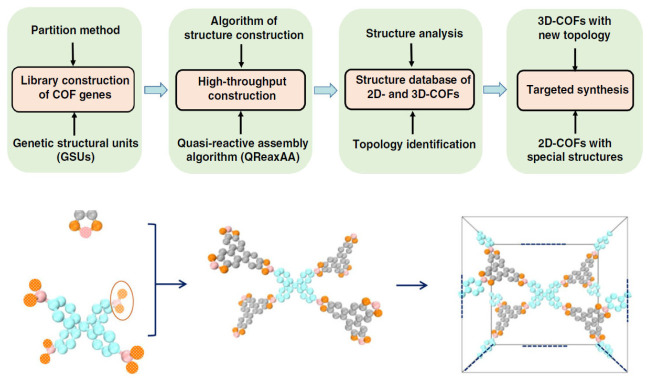
Schematic representation of the materials genomic method [40].

The original structures of the monomers are retained in the final structure of a 3D COF. Hence, genetic structural units (GSUs) are proposed as a method to mimic the natural formation of COFs as shown in Figure 7. Chemical knowledge and design are used for gathering genetic structural units from the already present components. Three rules have to be strictly followed during the partition method. Firstly, the binding sites should be strictly defined for the genetic structural units. Secondly, the integrity of the genetic structural units should be maintained. Thirdly, the reaction terminals should contain the reactive groups of the genetic structural units for particular reactions. Apart from the manipulation of material genes, the COF structures should be appropriately assembled by using an effective computer tool. A quasi-reactive assembly algorithms (QReaxAA) algorithm was used for the synthesis of a 3D COF [40]. The reactive points on the genetic structural units were bound using three different geometries by discarding the connections leading to high energy. The final geometry of the 3D COF was designed before its synthesis. 4,4′,4″,4‴-(ethene-1,1,2,2-tetrayl)tetraaniline (ETTA) was selected as a monomer for reacting with tris(4-formylphenyl) amine (TFPA) or 1,3,5-tri(4-formylphenyl)benzene to form 3D COFs with a ffc topology and noninterpreted structure. Hereafter, this method can be used for the synthesis of 3D COFs with topologies that are still not reported.

## 4. Topologies of 3D COFs Using Tetrahedral Units

### 4.1. The pts Topology

A pyrene-based 3D COF (3D-Py-COF) was synthesised to have a pts topology of two-fold points which can interpenetrate [24]. The crystal has a high surface area and narrow distribution of pores. In addition, it can selectively absorb carbon dioxide against nitrogen and exhibited fluorescent properties. The crystal was prepared by combining the rectangle (2D-C_2_) and tetrahedral (3D-T_d_) units through [4 + 4] condensation reaction of imines. The fluorescent property of the crystal was attributed to the isolated imine-functionalised pyrene moieties present in the 3D architecture. The presence of 20 ppm picric acid quenched its fluorescence properties.

Porphyrin and metal derivatives of porphyrin are macrocycles having extended π-electron conjugation with specific redox and photophysical characteristics [41]. A novel 3D porphyrin containing COF (3D Por-COF) was prepared using a tetrahedral (3D-T_d_) moiety by a [4 + 4] condensation reaction [42]. The crystals have large surface areas with a five-fold interpenetrated pts topology having a space group of Pmc2_1,_ and they produce oxygen in the singlet state under photoirradiation.

**Figure 8:**
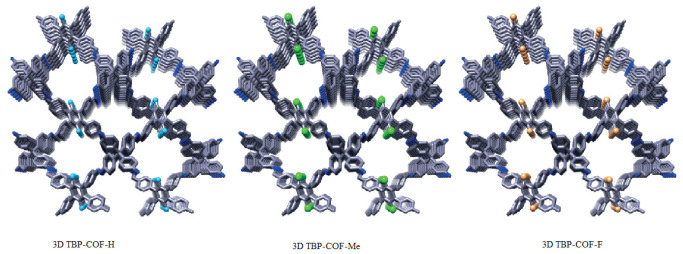
Geometrical representation of the structures [43].

Three isostructural 3D COFs were synthesised using the same skeleton but with different substituents (H, Me, and F) [43]. 1,2,4,5-tetraphenylbenzene (TPB) was selected as the quadrilateral core, and the 3- and 6-positions of the central phenyl ring were substituted by the three substituents to furnish three different COFs (3D TBP-COF-H, 3D TBP-COF-Me, and 3D TBP-COF-F) with pts topology as shown Figure 8.

There was a slight difference in the pore sizes of the three crystal types due to the volume effect since fluorine and methyl groups require larger areas as compared to a hydrogen atom. All three types of crystals absorbed carbon dioxide selectively from a mixture of gases containing carbon dioxide and nitrogen. A weak interaction between the COFs and nitrogen reduced the nitrogen uptake. However, the uptake of carbon dioxide was higher for 3D TBP-COF-F.

**Figure 9:**
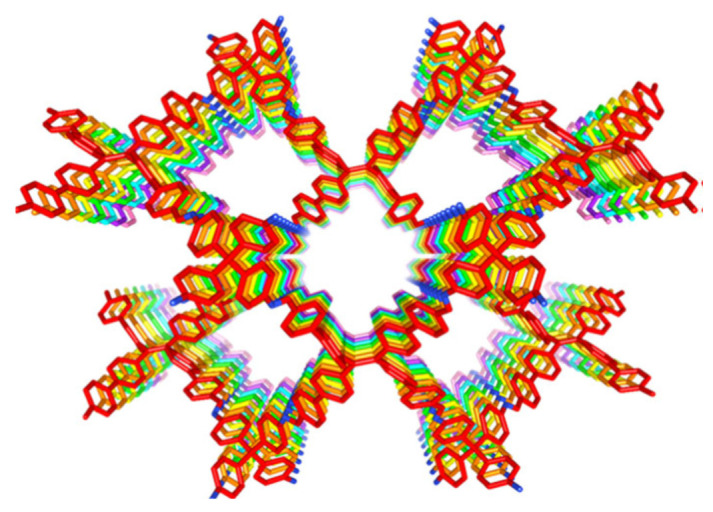
The porous structure of 3D-TPE-COF [44].

Tetraphenylethylene (TPE), an aggregation-induced emission luminogen (AIEgen), was used as a quadrilateral core to prepare AIEgen-based COFs (3D-TPE-COF) [44]. The photophysical phenomenon where aggregate luminogens are emissive while luminogens in dilute solutions are nonemissive is termed aggregation-induced emission (AIE) [45]. The 3D-TPE-COF had a large surface area with micropores, and a sevenfold interpenetrated pts topology as shown in Figure 9.

The crystal emitted fluorescent yellow light on excitation at 543 nm having a 20% quantum yield for photoluminescence. The aggregated state of tetraphenylethylene within the 3D framework has been ascribed to the fluorescence characteristics. The relaxation of tetraphenylethylene blocks is hindered by the presence of the rigid skeleton, thereby enhancing the fluorescence properties. The addition of picric acid quenched the fluorescence property. A white light-emitting diode was obtained by dip-coating a commercial blue light-emitting diode using 3D-TPE-COFs. The stability of the white light was up to 1200 h at room temperature. Thus, a white light-emitting diode can be fabricated by abstaining from the use of rare-earth metals. The morphology of the crystal was similar to a rod with the presence of microcrystals which are ordered over a long range. The crystal showed good carbon dioxide uptake capability. It was both chemically and thermally stable.

A tetrahedral geometry can be created from planar molecules by modulating the steric hindrance. The dihedral angle between two phenyl rings in biphenyls can be altered by introducing different substituents at the ortho position of the phenyl rings [46]. Thus, 3,3′,5,5′-tetra(p-aminophenyl)-bimesitylene (BMTA) was used as a linker to synthesise 3D COF-BMTA with an interpenetrated seven-fold pts topology [47]. The tetrahedral structure had a dihedral angle of 60°. Conjugation is present in the COF due to the presence of sp^2^ hybridised carbon atoms as a bridge in the nodes formed by biphenyls. Thus, the nonplanarity is due to steric hindrance aids in the design of different 3D COFs.

A woven 3D COF (COF-500-Cu) was prepared by interlocking 1-dimensional open square ribbons present in the shared corners of the squares by using rectangular tetratopic 4′,4‴,4⁗′,4⁗‴-(ethene-1,1,2,2-tetrayl)tetrakis(([1,1′-biphenyl]-4-amine)) (ETTBA) as linkers to connect Cu(PDB)_2_ with tetrahedral geometry. The topology of the crystal is similar to platinum sulphide (pts) [48]. The copper ions induce the accumulation of the square ribbons to form a 3D woven network. The crystallinity of the compound is retained even after the metal ion is removed and again remetalated later. Thus, the ribbons remain interlocked in the demetalated crystal but exhibited a high degree of freedom. Each of the macrocycles has an adjacent linker placed in its centre. The phenyl rings present in the linkers can move freely due to the separation between the corners of phenanthroline molecule and the linker. This phenomenon is responsible for fluorescence quenching. On demetalation, van der Waals force becomes maximum between the ribbons and the central linker. Hence, the rotation of the phenyl rings is hindered and emission induced by aggregation is reduced. The ribbons pack along the c-direction of the crystal densely. The demetalated COF exhibited good uptake of tetrahydrofuran and adopts a conformation similar to its metaled state.

### 4.2. Flexible 3D COFs

**Figure 10:**
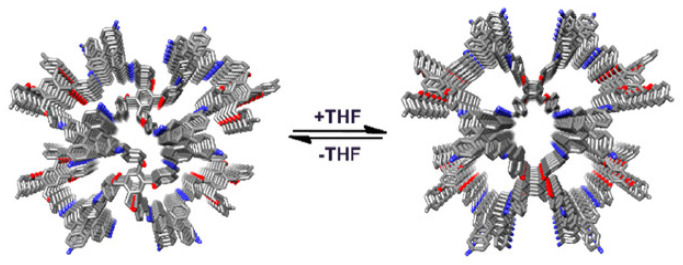
Flexible 3D COF in presence of an organic solvent Reprinted (adapted) with permission from [49]. Copyright 2021 American Chemical Society.

A flexible 3D COF (3D COF-flex) was synthesised with blocks 1,2,4,5-tetrakis[(4-formylphenoxy)methyl]benzene (TFMB) having a C-O single bond by using the method of continuous rotation electron diffraction (cRED) with a pts topology and six-fold interpenetration [49]. The skeleton exhibited a movement similar to breathing while absorption and desorption of tetrahydrofuran, an organic solvent, as shown in Figure 10. A composite film was fabricated using the crystal with PVDF to examine its application as a smart material. Thus, the film swelled and shrank in the presence and absence of tetrahydrofuran. A flower, fabricated using the composite, bloomed on exposure to tetrahydrofuran and returned to its original state in the air. Thus, this 3D COF can be used for the detection of solvents.

### 4.3. The dia Topology

Imine linkers were used for joining a linear unit, pyromellitic dianhydride (PMDA), and two different tetrahedral units, tetra(4-aminophenyl)methane (TAPM) and 1,3,5,7-tetraaminoadamantane (TAA) to form two 3D polyimide (PI)-based COFs (3D COF-PI-1 and 3D COF-PI-2) with a dia topology having four-fold interpenetrated nets [50]. The tetrahedral building units dictated the interpenetrated or non-interpenetrated geometry of the 3D COFs. The separation of the tetrahedral centres by long rod-shaped linkers formed the interpenetrated geometry. The crystal was investigated for delivering a drug molecule called ibuprofen. The drug uptake and release by both the COFs were good, which showed good promise as a drug transporter.

**Figure 11:**
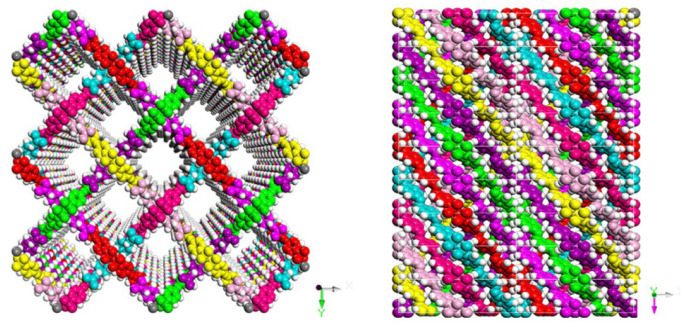
3D COF-DL229 with dia network [51].

A chemically stable and rigid porous framework was designed employing T_d_-symmetric adamantane knot to fabricate 3D COF-DL229 with dia topology [51]. The pore walls are formed by conjugated π-bonds along with ordered one-dimensional nanochannels to form a skeleton with an eight-fold interwoven structure as shown in Figure 11. The skeleton is flexible due to the existence of phenyl linkers at the edges where two imine linkages bind three phenyl groups. The channels are rectangular in shape and the edges are formed by stacking adamantane cores along the c-axis. Diphenylimine units are open to the channels. The crystals exhibited good capability to absorb iodine as the conjugated imine linkers and phenyl rings combine with iodine to form a charge-transfer complex. The iodinated complex has high conductivity due to the transfer of charges between channel walls of π-conjugation and iodine.

**Figure 12:**
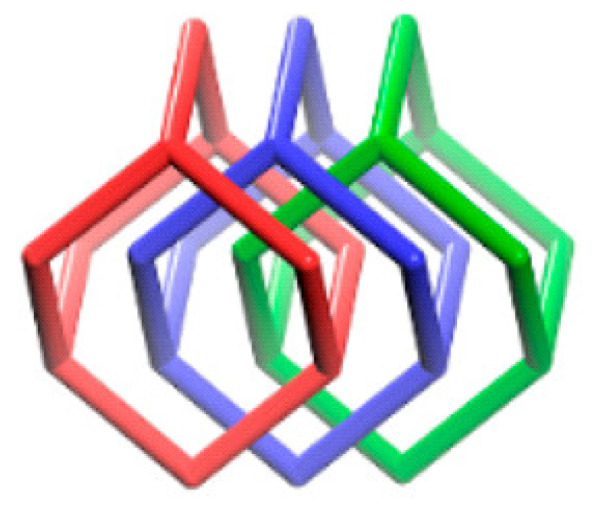
A 3-fold interpenetrated structure of 3D COF-anionic-DB. Reprinted (adapted) with permission from [52]. Copyright 2017 American Chemical Society.

Tetrahedral building blocks, tetrakis(4-formylphenyl)methane (TFPM), without any charge, were condensed with linear ionic bridging units, ethidium bromide (EB), and diimidium bromide (DB), to form positively charged COFs (3D COF-ionic-EB and 3D COF-ionic-DB) [52]. The COFs have a dia topology with three-fold interpenetrated assemblies, high porosity, selective ion exchange capability, and surface area. The linear bridging units have a node with two connections while the tetrahedral units have a node with four connections which combine to form a 3D dia topology as shown in Figure 12. The interaction due to the charged linear linkers and steric effect of both the linkers reduced the interpenetration. Methyl orange, a type of dye, was selectively captured by both crystals from a mixture of dyes containing methyl orange and methyl blue.

**Figure 13:**
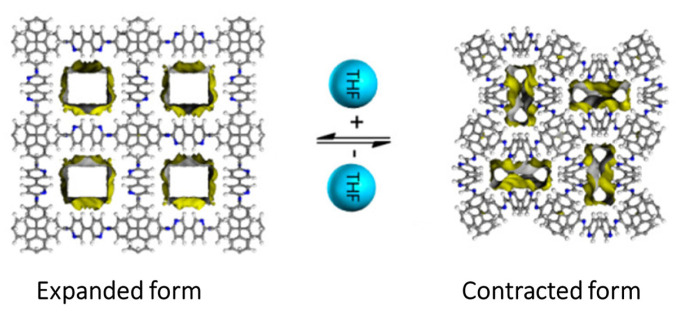
The flexible form of LZU-301. Reprinted (adapted) with permission from [53]. Copyright 2017 American Chemical Society.

An imine condensation of tetra(4-anilyl)methane (TAM) and (3,3′-bipyridine)-6,6′-dicarbaldehyde (BPyDA) furnished another 3D COF (LZU-301) having dia-C_9_-net topology [53]. The crystal has one-dimensional channels functionalized by pyridyl units. Their dynamic behaviour is because of the imine bonds moving around in the form of molecular pedals within the 3D architecture as shown in Figure 13. The crystals adsorbed carbon dioxide from both the humid and dry mixture of gases and catalyzed the Knoevenagel condensation reaction.

**Figure 14:**
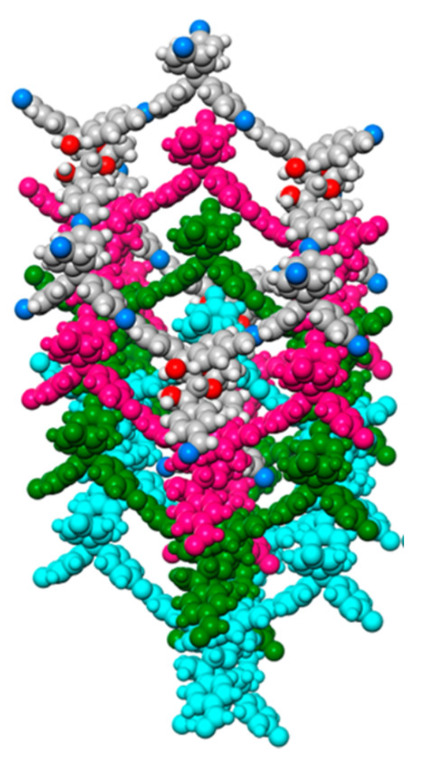
Fold interpenetration of a dia net along the a-axis. Reprinted (adapted) with permission from [54]. Copyright 2018 American Chemical Society.

A chiral 3D COF with a framework of amine linkage and dia topology was prepared by condensation of chiral tetraaryl-1,3-dioxolane-4,5-dimethanols (TADDOL) derived tetraaldehyde and tetrahedral tetra(4-anilyl)methane (TAM) [54] as shown in Figure 14. The chiral 3D COF is chemically stable and exhibited high porosity. The crystals have chiral tubular channels with apertures having sizes of 0.71 and 0.75 nm, respectively. These crystals are used for the chromatographic separation of enantiomers. The interior surface of the channels is amphiphilic and consists of chiral hydroxyl groups. These chiral hydroxyl groups are responsible for the interactions between alcohol groups and the host, leading to enantioselective separation during adsorption. The enantioselectivity was enhanced by oxidizing the imine to amide linkages for accurate modulation of the pores and improve the enantioselective resolution. Nevertheless, the resolution decreased and the chromatographic peak area increased with an increase in the mass of the injected racemic molecules.

**Figure 15:**
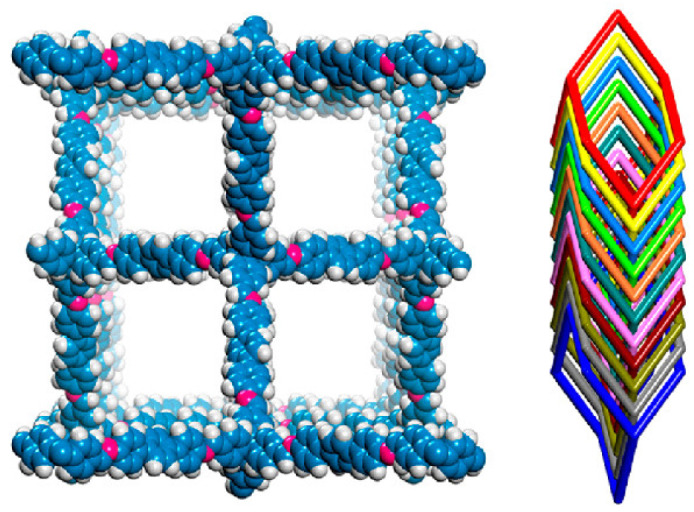
Structural presentation of 3D IL-COF. Reprinted (adapted) with permission from [55]. Copyright 2018 American Chemical Society.

An ionothermal synthesis of 3D COFs using ionic liquids (3D-IL-COFs) at room temperature and pressure within a short synthesis time (3 min) was reported. The crystals possess a topology of diamondoid (dia) nets [55] as shown in Figure 15. The ionic liquid 1-butyl-3-methylimidazolium bis((trifluoromethyl)sulfonyl)imide ([BMIm][NTf_2_]) was selected, and the tetrakis(4-formylphenyl)-methane (TFPM) acted as the tetrahedral building blocks. The ionic liquid reacted with three different molecules to furnish three different ionic liquid-based-COFs: 4,4′-diaminobiphenyl (DABP), p-phenylenediamine (PDA), and 4,4″-diamino-p-terphenyl (DATP). The 3D-IL-COF with 4,4″-diamino-p-terphenyl (DATP) showed 11-fold interpenetrated dia nets, while that with 4,4′-diaminobiphenyl (DABP) showed nine-fold interpenetrated dia nets. Alternately, p-phenylenediamine (PDA) synthesised a 3D-IL-COF with showed five-fold interpenetrated dia nets. All three 3D-IL-COFs showed good uptake of carbon dioxide from a mixture containing nitrogen and methane. The enhancement in the absorption of the gas selectively is attributed to the presence of ionic liquid in the pores of the crystals. The pores in ionic liquids retain carbon dioxide while methane and nitrogen filter out through the pores.

**Figure 16:**
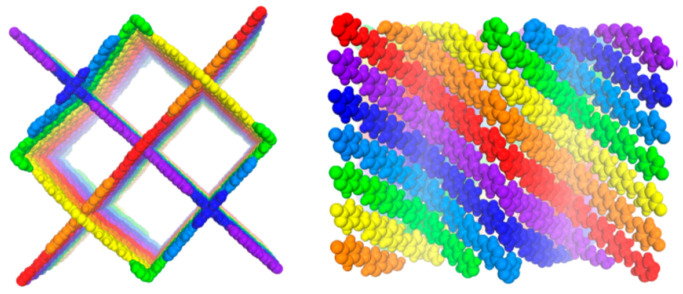
The structure of SP-3D-COF-1. Reprinted (adapted) with permission from [56]. Copyright 2018 American Chemical Society.

Spirobifluorene was used as the core building block for fabricating conjugated SP-3D-COFs with imine linkers [56]. Spirobifluorene was selected as the main linking unit due to its biplanar conjugated tetrahedral geometry. This molecule has the shape of a tetragonal disphenoid which is effective for forming tetrahedral nodes [57]. The presence of two rigid planar intermolecular fluorine units in an orthogonal configuration imparts stability to the 3D COF. Two different COFs were prepared by the condensation of 3,3′,6,6′-tetraamine-9,9′-spirobifluorene as a building group with 4,4′-biphenyldicarbaldehyde (A) and 1,4-phthalaldehyde (B) to form SP-3D-COF-A and SP-3D-COF-B, respectively, with a dia topology as shown in Figure 16. The normal orientation of C=N bond was present with a dia-c7 net and dia-c6 net for SP-3D-COF-A and SP-3D-COF-B, respectively. Multiple channels for transporting electrons were formed in a highly ordered sequence by the extended and rigid conjugated system entangled within the dia nets with high interpenetration degrees. Hence, these COFs were doped into CH_3_NH_3_PbI_3_ layers for enhancing the performance of perovskite solar cells.

In another study [58], a 3D COF (3D-HNU5) was designed using azine as a linker by condensation of (tetrakis(4-formylphenyl)-methane) and hydrazine in an ionic liquid [Bmim][Tf_2_N] (1-butyl-3-methylimidazolium bis((trifluoromethyl)sulfonyl)imide) at ambient temperature. The crystal possessed dia nets with a two-fold interpenetrated topology and is chemically and thermally stable. The crystal selectively absorbs carbon dioxide over nitrogen due to its porosity and narrow distribution of pore size. The skeleton was stable in the acidic solution but unstable in the alkaline solution as the reactions forming C=N gets reversed under alkaline conditions. Additionally, the crystal structure could not sustain very high alkaline and acidic conditions. The crystal was thermally stable even at 450 °C due to the existence of the conjugated and linear azine linker [59]. 3D-HNU5 contains excessive nitrogen atoms within the pores which lead to added Lewis acid–base interactions at low pressure and enhance the uptake of carbon dioxide. Moreover, the crystal can be recycled for up to five cycles without any loss in activity. The crystal was examined as support for Ag nanoparticles and used as a catalyst for the cyclisation of carbon dioxide with propargyl alcohols. The Ag-nanoparticles and carbon dioxide were dispersed within the crystalline pores which enhanced the conversion process.

**Figure 17:**
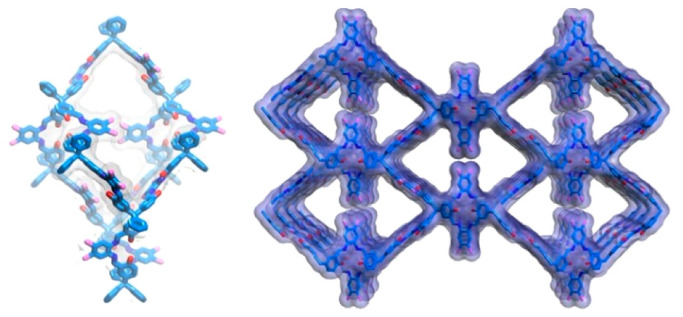
Structural representation of JUC-509. Reprinted (adapted) with permission from [60]. Copyright 2019 American Chemical Society.

A range of 3D Salphen-based COFs was designed and three different metal ions [Cu(II), Mn(II), and Eu(III)] were incorporated which exhibited good catalytic properties as antioxidants [60]. The Salphen moiety is obtained by the reaction between 4,5-dichlorophenylene-1,2-diamine (DCPDA) and 2-hydroxybenzaldehyde (HBA). Tetrakis(3-formyl-4-hydroxylphenyl) methane with a 4-connected node (TFHPM), a salicylaldehyde-based unit, was condensed with 4,5-dichlorophenylene-1,2-diamine with a 2-connected node to form JUC-509, and 4,5-difluorophenylene-1,2-diamine (DFPDA) with also a 2-connected node to obtain JUC-508. The COFs formed a non-interpenetrated dia topology having flower-shaped microcrystals as shown in Figure 17. The crystal structure was stable for the whole pH range with pores having a diameter of 1.2 nm. Agglomeration of COF crystals formed textural mesopores.

In another report, two 3D salen-based COFs were fabricated by the reaction between methane or silane derivatives of tetra-salicylaldehydes with ethanediamine to form a 7-fold interpenetrated dia topology [61]. These salen-COFs are used as a stationary phase in columns for separating xylene isomers and ethylbenzene, which is a challenging task because of similar boiling points [62], same sizes, and polarizability. Ethylbenzene was initially eluted from the column followed by p-xylene and m-xylene while o-xylene was eliminated as the final component. Both the COFs retained o-xylene for a longer duration due to the formation of weak bonds with the polar salen groups and both the methyl groups with a favourable geometry. However, the geometries are not advantageous for p- and m-xylene to interact with the salen groups and hence are eluted faster as compared to the other isomer. Moreover, the lowest value of dipole moment of ethylbenzene and p-xylene propels the molecules to be eluted faster. The amphiphilic surfaces of the channels having abundant polar salen groups of the 3D salen-based COFs govern the interactions between the guest and host molecules and impart selectivity to the column [63]. Additionally, the adsorption energy for edge-to-face stacking configuration of the isomers with salen-COFs is lower than that of face-to-face configuration. The configuration of edge-to-face is thermodynamically favoured.

**Figure 18:**
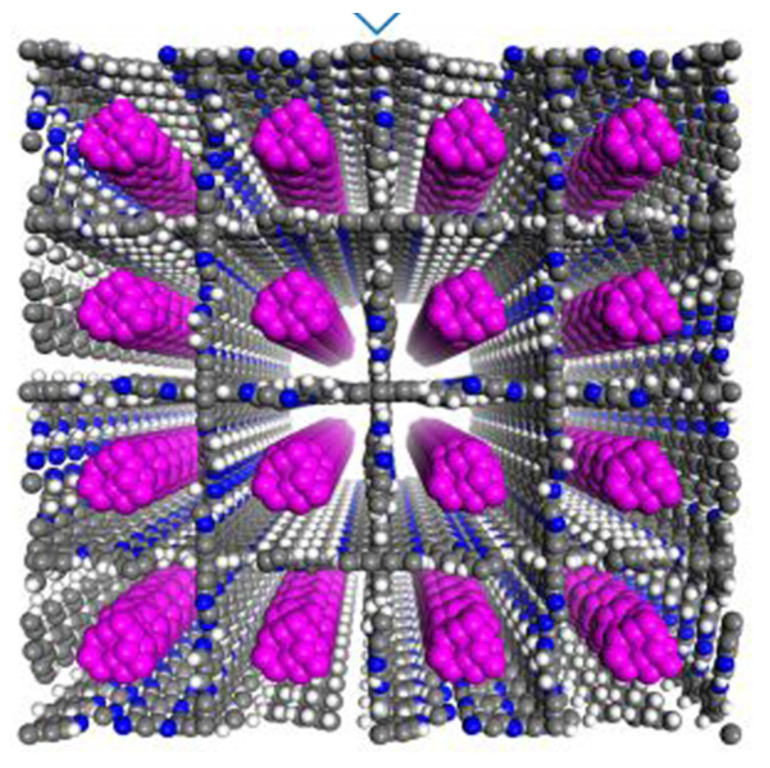
Schematic representation of 3D COF-SP-BPY [64].

Spirobifluorene with a tertragonal-disphenoid structure was used as a 3D building block to design 3D COFs (3D COF-SP) [64]. The COF has uniform one-dimensional channels because of the presence of two rigid and orthogonally intersected fluorine groups. 2,2′-bipyridine (BPY) units are used as linkers to form rigid channels with a 7-fold interpenetrated dia topology as shown in Figure 18. Bipyridine units formed chelation with Pd (II) metal and acted as a heterogeneous catalyst. The planar and rigid orthogonal spirobifluorene units formed a square and uniform channel with the pores. There are highly dense coordination points on the surface of the channel walls. The coordinated metal ions interact with the substrates effectively and behave as strong heterogeneous catalysts for the Suzuki–Miyaura reaction. The catalyst was recycled 5 times with little loss in its efficacy.

### 4.4. The ctn Net or bor Topology

**Figure 19:**
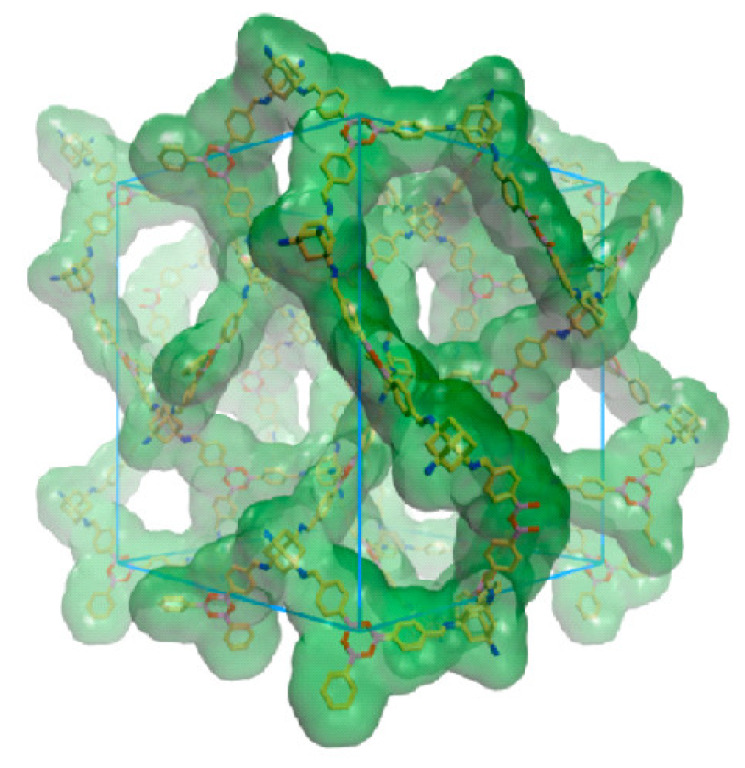
Structure of 3D DL-COF. Reprinted (adapted) with permission from [65]. Copyright 2016 American Chemical Society.

3D COFs having a cubic carbon nitride (ctn) net topology (3D-COF-FPBA and 3D-COF-FFPBA) were designed having two linking bonds, namely imine and boroxine with a huge capacity for storing carbon dioxide, methane, and hydrogen [65] as shown in Figure 19. Additionally, these crystals have a large surface area and are highly porous; they exhibit bifunctional cascade catalytic activity due to the presence of both basic and acidic sites. The morphology of the COF is rectangular with a size of 0.2 μm, and it is a highly potent heterogeneous catalyst because of the interconnected porous structure. Thus, acetal was hydrolysed by the basic sites of the imine group and acidic sites of boroxine groups.

### 4.5. ljh Topology

**Figure 20:**
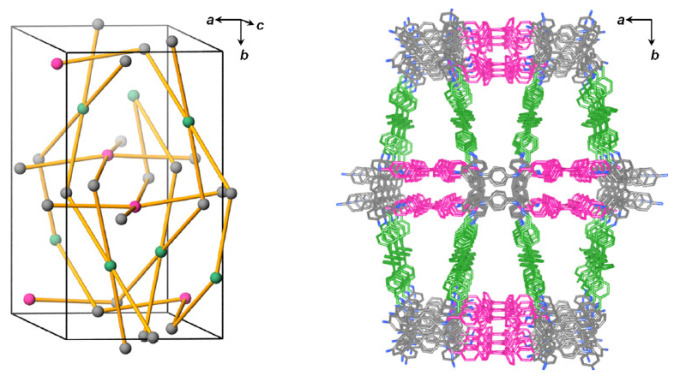
ljh Topology along c-axis. Reprinted (adapted) with permission from [66]. Copyright 2021 American Chemical Society.

A building unit with a bulky phenyl moiety, 1,2,4,5-tetrakis(4-formylphenyl)-3,6-diphenylbenzene (TPB-Ph) was condensed with tetra(p-aminophenyl)-methane (TAPM) to undergo [4 + 4] condensation to obtain a thermally stable 3D COF (3D COF-TPB-Ph) with an ljh topology [66]. Introducing two phenyl groups in 1,2,4,5-tetrakis(4-formylphenyl)-3,6-diphenylbenzene enhances the steric hindrance, which is minimum with the presence of two methoxy groups. Thus, the topology of the crystal altered from pts (with methoxy substituents) to interpenetrated ljh (with phenyl substituents) due to the strong impact between the pts nets lying adjacent to each other as shown in Figure 20.

### 4.6. rra Topology

**Figure 21:**
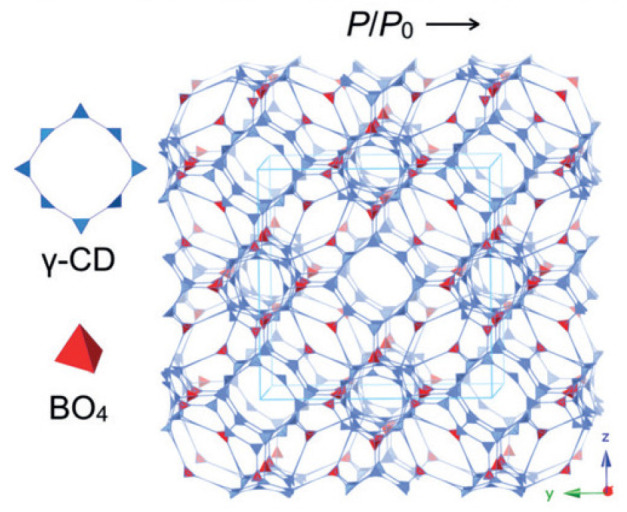
Synthesis of CD-COF-Li [67].

The 3D CD-COFs were fabricated using cyclodextrin as flexible building entities to obtain channels comprising dynamic functional groups [67]. Cyclodextrin is a cyclic oligosaccharide having a glucopyranose ring with secondary and primary hydroxy groups which act as joining units to create 3D polymeric architecture [68]. The transesterification reaction, a thermodynamically controlled reaction, between trimethyl borate [B(OMe)_3_] and hydroxyl groups was conducted to obtain an anionic tetrahedral structure, tetrakis(spiroborate) [69]. Thus, counterions are required to balance the negative charges and the selection of these counterions can be used to regulate the interactions with the approaching molecules [70]. Different counter ions were selected and γ-cyclodextrin was used as an organic support. This organic support was linked covalently using spiroborate as linkers. Three proton acceptors (Li^+^, piperazine, and dimethylamine) were examined for forming three different COFs (CD-COF-Li, CD-COF-PPZ, and CD-COF-DMA) with rra topology with high porosity as shown in Figure 21.

The nodes in the net are occupied by building blocks, where four γ-cyclodextrin units combine with one boron atom while eight boron atoms combine into a single γ-cyclodextrin moiety. The presence of channels for trapping electrolytes and an anionic skeleton makes CD-COF-Li a promising Li-ion conductor in the solid state. An enhancement in temperature decreases the resistance of the material. The cell fabricated of this COF exhibited good cyclic stability since the polarization effect is diminished due to the reduction of the concentration gradients of anions by the porous anionic geometry. This also minimizes the formation of Li dendrites. Among the three different COFs, CD-COF-PPZ showed the highest absorption capacity of CO_2_ due to the presence of protonated piperazine around the pores that enhances the attraction for CO_2_ molecules through resilient quadrupole moments.

### 4.7. Topologies of 3D COFs Using Tritopic Building Blocks: srs Topology

**Figure 22:**
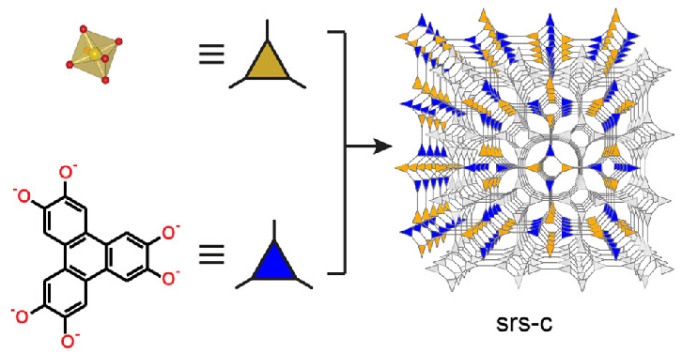
Structure of SiCOF-5 net. Reprinted (adapted) with permission from [68]. Copyright 2018 American Chemical Society.

In one study, a 3D anionic COF-containing Si (3D Si-COF-5) was synthesised having an srs topology as shown in Figure 22 [68].

However, the preparation of the crystal was challenging as simple condensing of tetramethoxysilane (TMOS) or silica gel and 2,3,6,7,10,11-hexahydroxytriphenylene (HHTP) failed to generate the crystal. The rate of the reaction can be modulated by controlling the amount of water, nucleation, growth, and solubility of linkers in the reaction system. Thus, the source of silicon was controlled in situ to propel the crystallization of 3D SiCOFs. Methyltrimethoxysilane was disproportionated in the presence of nucleophiles (alkali metal methoxide as bases) to form tetramethoxysilane and tetramethylsilane in situ.

The different COFs based on topology and pores size were summarized in Table 1. A larger surface area, good confinement effect, and abundant open sites in 3D COFs as compared to those of 2D COFs make them promising materials for various applications. The common technique to control the porosity of COFs is to improve interlayer interactions and self-complementary π-electronic forces. 

According to computational investigations, the self-complementary π-electronic force increases the overall crystal stacking energy while minimizing the unit cell size, which improves the porosity of COFs [72].

## 5. Application of 3D COFs as Catalyst

**Figure 23:**
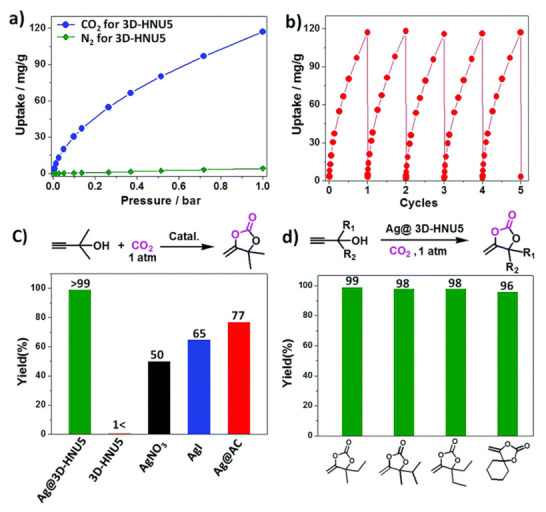
(**a**–**d**) The conversion and capture of CO_2_ using azine-linked 3D COF [58].

Pengxin and co-workers [58] revealed that Ag nanoparticles loaded into azine-based 3D COFs are good heterocatalysts for the transformation of carbon dioxide into different compounds with a 99% yield, as shown in Figure 23. The Ag nanoparticles are highly dispersed in the pores of the crystals which improved the catalytic property. This heterogeneous catalyst also catalyzed the reaction of carbon dioxide with a variety of reactants. Another series of anionic cyclodextrin-based 3D COF exhibited good uptake of carbon dioxide [67]. The presence of piperazine as a proton acceptor in the 3D COF-CD-PPZ enhances the gas uptake. This is ascribed to the fact that the pores of the crystal are occupied by the protonated piperazine that boosts the carbon dioxide affinity through relatively strong quadrupole moments. The physical adsorption (1.59 mmol g^−1^ at 298 K) of carbon dioxide was confirmed by pyridyl-based 3D COFs [53]. The retention time of the gas was 25 min under dry conditions which increased substantially under wet conditions.

**Figure 24:**
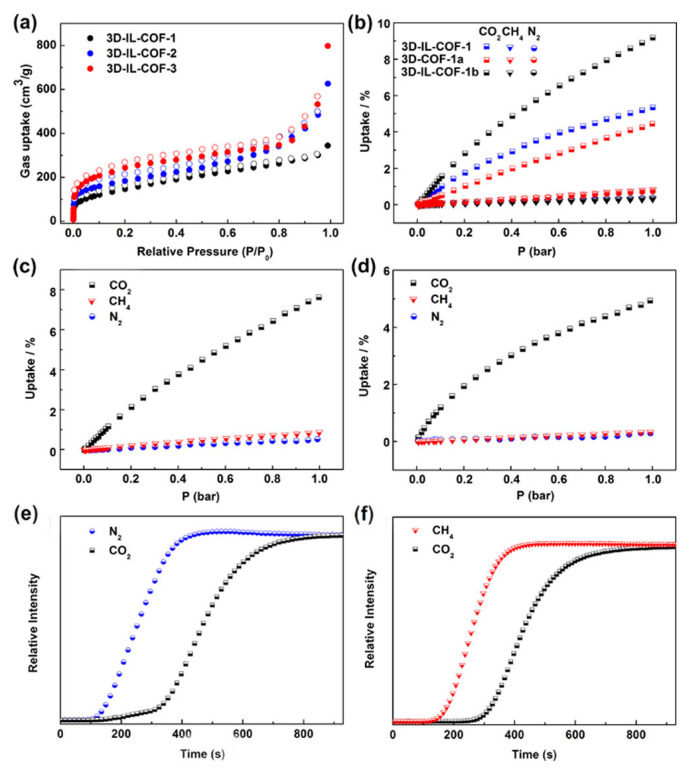
(**a**–**f**) The impressive presentation in the separation of CH_4_/CO_2_/N_2_ using 3D-IL-COFs. Reprinted (adapted) with permission from [55]. Copyright {2018} American Chemical Society.

Additionally, the uptake was further enriched in the presence of moisture [73]. 1,2,4,5-tetraphenylbenzene-based 3D COFs were found to selectively uptake carbon dioxide from a mixture of gases containing nitrogen and carbon dioxide [46]. This is because the quadrupole moment of carbon dioxide is higher than that of nitrogen. The amount of sorption can be modulated by tuning the pore structures of these COFs. Ionic liquid-based 3D COF also confirmed a good uptake of carbon dioxide from a mixture containing carbon dioxide, nitrogen, and methane [55], as shown in Figure 24.

A good uptake of carbon dioxide was observed for an azine-based 3D COF due to the presence of abundant nitrogen atoms within the pores of the crystal [58]. Thus, Lewis acid–base interactions are highly boosted under low pressure. Moreover, the crystal had good reusability with no loss in adsorption as 100% carbon dioxide adsorption was observed after five rounds.

## 6. Conclusions

We have summarized our review with a growth mechanism and topologies of 3D COFs using tritopic building blocks and tetrahedrals units. Additionally, there is heavy demand for new topologies. The determination of 3D COF structures is a challenge due to the difficulty of obtaining a single crystal of large size. The 3D COFs are obtained as polycrystalline materials. Hence, techniques are essential for obtaining a large single crystal for the synthesised 3D COFs. The lack of additional propelling forces, such as π–π stacking, hinders the formation of crystals and initiates the formation of amorphous frameworks.

The majority of the reported 3D COFs have interpenetrated structures with srs, pts, and dia topologies. An increase in the interpenetration number decreases the pore volume, specific surface area, and pore size of 3D COFs. Interconnected 3D channels are present in less or non-interpenetrated frameworks. High-interpenetrated channels contain interconnected 1D channels. The optimization of monomers and conditions for synthesis can modulate interpenetration. There requires a study for searching suitable monomers for specific topologies to reduce interpenetration. The development of more stable COFs is compulsory.

The 3D COFs have potential applications based on their remarkable properties: high surface areas, interpenetrated channels, pore environment that can be manipulated, good chemical stability, and abundant active sites which are accessible. Numerous 3D COFs with different functional groups have been synthesised. However, the synthesis of 3D COFs is difficult. The funtionalisation and crystallization of 3D COFs require further research. Different synthetic methods of 3D COFs have been reported: ionothermal, solvothermal, microwave-assisted, and amorphous transformation methods.

Hence, work is required for the development of facile synthetic methods (sonochemical synthesis, electrochemical synthesis, mechanochemical synthesis, and electrochemical synthesis) for 3D COFs which are not possible to obtain through conventional methods.

## Figures and Tables

**Table 1:** The comparative properties of 3D COFs.

Type of 3D COFs	Topology	Pore Size	BET Surface Area m^2^ g^−1^	Pore Volume cm^3^ g^−1^	Ref
LZU-301	dia-C9	5.8 × 10.4 Å^2^	654		[53]
3D Por-COF	pts	0.60 nm	1398		[42]
3D CuPor-COF	pts	0.63 nm	1335		
3D TPB-COF-H	pts		1050	0.49	[43]
3DTPB-COF-Me	pts		950	0.45	
3D TPB-COF-F	pts		850	0.42	
Chiral-COF	dia	0.62 nm	655	0.51	[54]
3D IL-COF-PDA	dia	8.3 Å	517	0.36	[55]
3D IL-COF-DABP	dia	10.7 Å	653	0.53	
3D IL-COF-DATP	dia	12.4 Å	870	0.56	
SP-3D-COF-2	dia		641	0.45	[56]
SP-3D-COF-1	dia		1582	0.97	
3D-TPE-COF	pts		1084	0.55	[44]
3D-HNU5	dia		864	0.89	[58]
JUC-508	dia	1.3 nm	1513		[60]
JUC-509	dia	1.2 nm	1443		
3D COF-salen-Si	dia	7.8 Å	666	0.43	[61]
3D COF-salen-Me	dia	7.8 Å	701	0.38	
3D COF-SP-BPY	dia	1.36 nm	1945	1.10	[64]
3Dchiral-COF LZU-111	lon-b-c3	10.9 Å	2120	0.92	[50]
3D COF-500-Cu	pts	12.6 Å	352		[60]
3D LZU-306	pts	10.9 Å	2059		[51]
3D cage-COF	acs	8.8 Å, 5.6 Å	1040		[32]
3D cage-COF-H	acs	10.5 Å, 11.4 Å	1143		[71]
3D cage-COF-OH,	acs	6.5 Å	923		
3D cage-COF-Cl	acs	5.3 Å	660		
